# Clinical, Laboratory, and Molecular Characteristics of *GPD1* Gene Variants: A Cause of Hepatomegaly and Hepatic Steatosis in Early Childhood

**DOI:** 10.1155/grp/9986415

**Published:** 2026-04-09

**Authors:** Moodhi Alharbi, Ali Alasmari, Sultan Alkasim, Abdulrahman Al-Hussaini

**Affiliations:** ^1^ Division of Pediatric Gastroenterology, Maternity and Children′s Hospital, Buraydah, Saudi Arabia, moh.gov.sa; ^2^ Division of Medical Genetics, The Children′s Hospital, King Fahad Medical City, College of Medicine, King Saud bin Abdulaziz University for Health Sciences, Riyadh, Saudi Arabia, ksau-hs.edu.sa; ^3^ College of Medicine, King Saud University, Riyadh, Saudi Arabia, ksu.edu.sa; ^4^ Division of Pediatric Gastroenterology, Children′s Specialized Hospital, King Fahad Medical City, Riyadh, Saudi Arabia, kfmc.med.sa; ^5^ College of Medicine, Alfaisal University, Riyadh, Saudi Arabia, alfaisal.edu; ^6^ King Saud University Celiac Disease Research Chair, Department of Pediatrics, Faculty of Medicine, King Saud University, Riyadh, Saudi Arabia, ksu.edu.sa

**Keywords:** cirrhosis, *GPD1* gene, hepatic steatosis, hypertriglyceridemia, Saudi Arabia

## Abstract

**Background and Objectives:**

Mutations in the *GPD1* gene, which encodes glycerol‐3‐phosphate dehydrogenase 1 (GPD1), are a rare cause of monogenic hypertriglyceridemia (HTG) during childhood. This study is aimed at providing a detailed analysis of the clinical, laboratory, and molecular characteristics of all patients affected by biallelic *GPD1* gene variants, including three cases from our center.

**Methods:**

A literature search was conducted across the English MEDLINE, PubMed, and Embase databases from 1966 to 2023 for the studies that reported on GPD1 deficiency with confirmed *GPD1* gene variants using the search words HTG and *GPD1* gene.

**Results:**

A total of 39 children (including three from our center) were identified with 15 pathogenic mutations in *GPD1* reported in 14 articles from 5 ethnicities (15 Arabs, 14 Caucasians, 4 Chinese, 5 Indian/South Asian, and 1 Turkish): 8 missense variants (one compound heterozygous), 3 nonsense, 3 frameshifts, and 1 splicing. Three of the 15 variants likely originated from a common ancestor, “that is, founder in nature”: p.I119fs∗94 (Palestinian Arabs), p.Gly299Arg (Czech population), and p.Thr251Asnfs∗10 (Saudi Arabia). The median age at presentation was 9 months. All patients manifested hepatomegaly, elevated transaminases, and HTG. Nineteen patients underwent liver biopsy; all showed micro‐ and macrosteatosis, mild to moderate portal fibrosis in 58% (11 out of 19), and cirrhosis in the remaining eight cases (42%). Follow‐up data showed that HTG normalized in 28% of patients (11 out of 39) but persisted mildly in the remaining 72% (28 out of 39).

**Conclusion:**

Mutations in the *GPD1* gene should be considered in children with hepatomegaly, hepatic steatosis, and HTG. This study indicates that *GPD1* deficiency may not be a transient or benign condition, as previously considered, due to the persistence of HTG and liver pathology in a significant proportion of cases. Identifying specific GPD1 variants may expedite targeted molecular analysis.

## 1. Introduction

Mutations in the *GPD1* gene cause a rare autosomal recessive disorder labelled as “transient infantile hypertriglyceridemia (HTGTI).” This disease is relatively a newly discovered monogenic form of alteration in lipid metabolism; the first report on *GPD1* mutations was in 2012, when Basel‐Vanagaite et al. reported on 10 family members from 4 consanguineous Arab families in Palestine [1]. The typical clinical manifestations were transient hypertriglyceridemia (HTG), hepatomegaly, elevated liver transaminases, and hepatic steatosis in early infancy. Later, additional similar and even more heterogeneous phenotypes have been reported (including recurrent hypoglycemia, obesity, kidney disease, and short stature) in different countries of the world and among various ethnic groups [1–14].

Lipid metabolism disorders, like HTG, arise from the intricate interplay between genetic factors and metabolic pathways. Elevated TG levels are generally sourced from either exogenous TG (absorbed in the gut and transported via chylomicrons) or endogenous TG (synthesized in the liver and secreted in very low–density lipoprotein [VLDL] particles). Normally, these processes are tightly regulated by enzymes, apolipoproteins, and transport pathways that help maintain TG balance [15].

The *GPD1* gene encodes the intracellular isomer of glycerol‐3‐phosphate dehydrogenase 1 (GPD1), which is crucial in lipid metabolism. It catalyzes the conversion of dihydroxyacetone phosphate (DHAP) and reduced nicotine adenine dinucleotide (NADH) to glycerol‐3‐phosphate (G3P); thereby, deficiency of GPD1 leads to an accumulation of DHAP [1]. Excessive amounts of DHAP may cause increased fat synthesis in the acyl DHAP pathway. Therefore, *GPD1* mutations lead to increased TG synthesis in the liver, decreased output from the liver, increased inflow of fatty acids into the liver, and impaired hepatic beta‐oxidation causing nonalcoholic hepatic steatosis [1].

This article incorporates three Saudi pediatric patients with a novel *GPD1* gene variant to the 36 genetically confirmed patients previously reported in 14 case reports/series [1–14], and provides a detailed analysis of the clinical, laboratory, and molecular characteristics of all the 39 patients affected with biallelic *GPD1* gene variants.

## 2. Methods

We searched the English MEDLINE, PubMed, and Embase databases from 1966 to 2024 for the studies that reported on HTGTI cases with confirmed *GPD1* gene variants using the search words HTGTI and *GPD1* gene. We found 15 studies and excluded one study that was in a non‐English language [16]. Rest of the 14 studies were reviewed for demographics, clinical presentation, ethnicity, laboratory parameters (triglyceride level [TG], alanine aminotransferase [ALT], aspartate aminotransferase [AST], and *γ*‐glutamyl transferase [GGT]) at the presentation and follow‐up, imaging findings, the result of molecular test and type of *GPD1* variant, and histopathological features on liver biopsies.

This study was conducted in accordance with the Declaration of Helsinki and approved by the Institutional Review Board (IRB) of King Fahad Medical City, Riyadh, Saudi Arabia (Number 23‐241).

## 3. Results

A total of 39 children (17 males and 22 females), including three from our center, were identified with pathogenic mutations in *GPD1* reported in 14 articles from different populations (15 Arabs, 14 Caucasians, 4 Chinese, 5 Indian/South Asian, and 1 Turkish). The epidemiologic, clinical, laboratory, and molecular features of individuals with biallelic mutations in the GPD1 gene are summarized in Table 1.

**Table 1:** Clinical, laboratory, and molecular characteristics of 39 patients with GPD1 deficiency.

References	Case	Descent	Onset age (month)	Sex	Mutations in *GPD1* gene	Variant type	Elevated TG	Last TG	Elevated ALT/AST	Last ALT/AST	Hepatomegaly	Steatosis	Liver biopsy	Other features
Basel‐Vanagaite et al. [1]	1	Arab	1	M	c.361 − 1G > C, p.I119fs∗94	Splicing	+	↑	+	−	+	+	ND.	Vomiting
	2	Arab	1	M	c.361 − 1G > C, p.I119fs∗94	Splicing	+	↑	+	↑	+	+	Portal fibrosis.	Vomiting
	3	Arab	4–6	M	c.361 − 1G > C, p.I119fs∗94	Splicing	+	↑	+	N	+	+	Portal fibrosis.	Short stature
	4	Arab	At birth	F	c.361 − 1G > C, p.I119fs∗94	Splicing	+	N	+	↑	+	+	ND.	Short stature
	5	Arab	6	F	c.361 − 1G > C, p.I119fs∗94	Splicing	+	↑	+	↑	+	+	ND.	Failure to thrive
	6	Arab	2.5	F	c.361 − 1G > C, p.I119fs∗94	Splicing	+	N	+	−	+	+	ND.	Vomiting
	7	Arab	7	F	c.361 − 1G > C, p.I119fs∗94	Splicing	+	N	+	↑	+	+	ND.	—
	8	Arab	7	M	c.361 − 1G > C, p.I119fs∗94	Splicing	+	↑	+	↑	+	+	ND.	—
	9	Arab	9	M	c.361 − 1G > C, p.I119fs∗94	Splicing	+	↑	+	↑	+	+	ND.	Short stature; horseshoe kidney
	10	Arab	3.5	F	c.361 − 1G > C, p.I119fs∗94	Splicing	+	↑	+	↑	+	+	ND.	Short stature; craniocerebral involvement

Joshi et al. [2]	11	Caucasian	5	F	c.686G > C, p.R229P	Missense	+	↑	+	NA	+	+	Portal fibrosis.	Small head circumference; failure to thrive; vomiting
Dionisi‐Vici et al. [3]	12	Arab	10	F	c.806G > A, p.R269Q	Missense	+	N	+	N	+	+	ND.	Fasting hypoglycemia
	13	Caucasian	12	M	c.361 − 1G > C, p.I119fs∗94	Splicing	+	N	+	NA	+	+	Early cirrhosis.	
	14	Caucasian	5	M	c.640T > C, p.C214R	Missense	+	↑	+	↑	+	+	Cirrhosis.	Renal disease, TBA elevation; dicarboxylic aciduria
	15	Caucasian	24	M	c.640T > C, p.C214R	Missense	+	↑	+	↑	+	+	Mild portal fibrosis.	At last F/U, 31 years is doing well, mild hepatomegaly

Li et al. [4]	6	Chinese	84	M	c.220‐2A>G; c.820G > A, p.A274T	Missense	No	NA	No	N	NA	+	ND.	Short stature; obesity; insulin resistance; dermal abnormalities; EDL
Li et al. [5]	17	Chinese	3.5	F	c.523C > T, p.Q175∗	Nonsense	+	↑	+	↑	+	+	Mild Portal fibrosis.	TBA elevation
Matarazzo et al. [6]	18	Caucasian	12	M	c.895G > A, p.G299R	Missense	+	↑	+	↑	+	+	Cirrhosis.	TBA elevation
Wang et al. [7]	19	Chinese	5 days	F	c.901 G > T (p.E301X)	Missense	+	N	+	↑	+	+	ND.	Indirect hyperbilirubinemia
Lin et al. [8]	20	Chinese	4	M	c.454C > T, p.Q152∗	Nonsense	+	N	+	N	+	+	ND.	TBA elevation; failure to thrive; short stature
Kumar et al. [9]	21	Indian	2	M	c.500G > A (p.Gly167Asp)	Missense	+	NA	No	NA	+	+	Portal fibrosis.	—

Tesarova et al. [10]	22	Caucasian	NA	NA	c.895G > A (p.Gly299Arg)	Missense	+	NA	+	NA	+	Liver biopsy performed in six patients revealed micro/macrovesicula and even cirrhosis in two boys.		Two infants developed mild hypoglycemia.
	23	Caucasian	NA	NA	c.895G > A (p.Gly299Arg)	Missense	+	NA	+	NA	+			
	24	Caucasian	NA	NA	c.895G > A (p.Gly299Arg)	Missense	+	NA	+	NA	+			
	25	Caucasian	NA	NA	c.895G > A (p.Gly299Arg)	Missense	+	NA	+	NA	+			
	26	Caucasian	NA	NA	c.895G > A (p.Gly299Arg)	Missense	+	NA	+	NA	+			
	27	Caucasian	NA	NA	c.895G > A (p.Gly299Arg)	Missense	+	NA	+	NA	+			
	28	Caucasian	NA	NA	c.895G > A (p.Gly299Arg)	Missense	+	NA	+	NA	+			
	29	Caucasian	NA	NA	c.895G > A (p.Gly299Arg)	Missense	+	NA	+	NA	+			
	30	Caucasian	NA	NA	c.895G > A (p.Gly299Arg)	Missense	+	NA	+	NA	+			
	31	Arab	NA	M	c.116G > A (p.Trp39∗)	Nonsense	+	NA	+	NA	+			

Kumar et al. [11]	32	Indian	7	M	c.500G > A (p.Gly167Asp)	Missense	+	↑	+	↑	+	+	Early cirrhosis	Hepatic adenoma, hypoglycemia.
Polchar et al. [12]	33	South Asian	23	F	c.500G > A (p.Gly167Asp)	Missense	+	N	+	↑	+	+	Early cirrhosis	Growth and developmental delay.
Gunes et al. [13]	34	Turkish	3	M	c.936_940del (p.His312GlnfsTer24)	Frameshift	+	↑	+	N	+	+	ND	Severe anemia requiring multiple blood transfusion.
Sharma et al. [14]	35	Indian	1.5	F	p.Val194GlyfsTer22	Frameshift	+	↑	+	↑	+	+	Portal fibrosis	Obstetric history: two mid‐trimester abortions, one stillbirth.
	36	Indian	15	M	p.Val194GlyfsTer22	Frameshift	+	↑	+	↑	+	+	Septal fibrosis	Growth retardation.

Alharbi et al. (present cases) [17]	37	Arab	12	M	c.751dup p.(thr251Asnfs∗10)	Frameshift	+	↑	+	↑	+	+	ND	Incidental finding during investigation for bronchiolitis.
	38	Arab	7	M	c.751dup p.(thr251Asnfs∗10)	Frameshift	+	↑	+	↑	+	+	ND	Presented with abdominal distention.
	39	Arab	29	F	c.751dup p.(thr251Asnfs∗10)	Frameshift	+	↑	No	NA	+	+	ND	Incidental finding during admission for septic arthritis.

### 3.1. The Demographic, Epidemiologic, and Clinical Characteristics

The median age at disease onset was 9 months (range: birth–84 months). A total of 48% of the patients (13 out of 27) were discovered incidentally during evaluation for another illness, whereas two patients were identified through family screening due to a positive family history of *GPD1* mutations. The most common clinical findings were hepatomegaly (100%), growth impairment in 12 patients, hypoglycemia in 3 [3, 10], and 1 case involving obesity, short stature, and insulin resistance [4]. In our cohort, hepatomegaly and elevated liver transaminases were identified during evaluation for bronchiolitis, septic arthritis, and abdominal distention.

### 3.2. Laboratory Data

At initial presentation, all 39 patients demonstrated elevated liver transaminases, with AST levels higher than ALT levels. Elevated GGT and serum TGs were also universally observed.

### 3.3. Histopathological Findings

Nineteen patients underwent liver biopsy; all manifested with micro‐ and macrosteatosis, mild to moderate portal fibrosis in 58% (11 out of 19), and cirrhosis in the remaining cases, 42% (8 out of 19).

### 3.4. Molecular Analysis

Fifteen *GPD1* variants were identified in five major ethnicities: eight missense variants (one compound heterozygous), three nonsense, three frameshift, and one splicing. Three of the 15 variants probably came from a common ancestor, “that is, founder in nature”: p.I119fs∗94 (Palestinian Arabs), p.Gly299Arg (Czech population), and p.Thr251Asnfs∗10 (Saudi Arabia). The p.Thr251Asnfs∗10 variant was identified in two unrelated families from two main tribes in Saudi Arabia, that have not been reported in other populations, indicating that this mutation probably came from common origin that is, “founder gene mutations.”

### 3.5. Follow‐Up and Treatment

The average follow‐up period across all cases was 7 years, with a range from 11 months to 31 years. Follow‐up assessments were available for 29 of the 39 cases. Among the 29 patients with follow‐up data, TG levels remained elevated in 19 cases (66%), normalized in 8 cases (28%), and were unavailable in 2 cases (7%).

Lipid‐lowering therapy was administered to three patients, including one from our cohort. Among these treated cases, one patient demonstrated complete normalization of TG levels and improved liver elastography values, with a reduction from 11.5 to 10.6 kPa after 1 year of therapy.

## 4. Discussion

To date, 39 genetically confirmed patients have been reported worldwide (including 16 *GPD1* variants) in 15 case reports and series, including our paper. This is the first report of a *GPD1* pathologic variant identified in Arabs outside of the Arab population in Palestine; we have expanded the spectrum of mutations in the *GPD1* gene to include a novel frameshift founder mutation and the geographic distribution to include the Arabian Peninsula. Our review of the 39 patients with biallelic *GPD1* variants, including our cases, highlights several important observations. First, typically all the cases presented with hepatomegaly, elevated transaminases, HTG, and liver steatosis, predominantly during infancy (90%) and usually as incidental findings during evaluation for another illness or screening other siblings in the family. Second, although the severe HTG during infancy normalized in only 30% of the patients on follow‐up, the elevated TG levels persisted in the mild to moderate range in the remaining 70%. Third, the liver steatosis subsequent to HTG led to persistent inflammation and a mild to moderate grade of portal fibrosis in 58% and cirrhotic changes in 42% of the 19 liver biopsies obtained during infancy. These observations are in contrast to the perception that HTG is transient and the disease course is benign, which have been falsely understood from the original description of the disease “HTGTI,” which led many investigators to propose the name GPD1 deficiency or *GPD1* gene mutation instead.

The natural course and long‐term prognosis of GPD1 deficiency is not clearly defined. Some patients persisted to have mild to moderate HTG until the age of 23 and 31 years with normal growth and nonprogressive liver disease [1, 3]. In contrast, other patients developed cirrhosis during infancy with insufficient data on long‐term follow‐up. Further longitudinal studies are required to determine the long‐term prognosis of the liver disease and the potential predictors of worse outcomes. Based on the available limited data, the severity of the clinical phenotype of *GPD1* mutation‐related disease did not correlate with the type of mutation variant. Even within a single family with the same *GPD1* variant, the severity of the disease was variable; an infant (Patient Number 14 in Table 1) had cirrhosis on liver biopsy performed at the age of 5 months, and the uncle (Patient Number 15) had mild portal fibrosis on liver biopsy at the age of 2 years and is doing very well at the age of 31 years with mild elevation of liver transaminases and TG levels.

Another still unclear aspect of GPD1 deficiency is the negative impact of persistent HTG on the health. HTG is a well‐recognized risk factor for atherosclerosis, coronary heart disease, and acute pancreatitis [15, 18–20]. According to the medium follow‐up data from patients with *GPD1* mutations, no evidence of coronary heart disease or pancreatitis was reported, not even in the 23 and 31‐year‐old patients [1, 3]. However, most of the patients reported so far are young, and no conclusions can be drawn on the negative impact of severe HTG during childhood on the risk for coronary heart disease or pancreatitis. Therefore, it is necessary to follow up the GDP1 deficient patients closely in the long‐term. Is medical or dietary therapy of the HTG secondary to DPG1 deficiency during childhood necessary to prevent progression of the liver disease and other morbidities in the long‐term? At present, there is no evidence to support the regular use of lipid‐lowering agents in patients with GPD 1 deficiency and data on the efficacy and safety are lacking. We and others [6] have successfully used lipid‐lowering drugs to reduce markedly elevated serum triglycerides. These drugs and/or dietary modification (low‐fat diet) could represent a possible therapeutic option for some patients specially with those with persistent marked elevation of triglyceride levels (triglyceride level of > 500 mg/dL) [21] or in patients with evidence of liver fibrosis. Long‐term follow‐up multicenter trials of infants and children with GDP1 deficiency might help answer this question.

The novel *GPD1* variant (p.Thr251Asnfs∗10) identified in our population is harbored by two unrelated families from two main tribes that include more than 1 million people. The prevalence of consanguineous marriage in Saudi Arabia is almost 60% [22], which presents a major risk factor for autosomal recessive disease such as GPD1 deficiency. Before writing up our present three cases, we contacted all the major tertiary care centers in Saudi Arabia and, to our surprise, no cases of GPD1 deficiency were diagnosed in any of these centers. This raises the possibility of the underdiagnosis of GPD1 deficiency in Saudi Arabia. The phenotypic spectrum of GPD1 deficiency (hepatomegaly, hepatic steatosis, HTG, elevated liver enzymes, and hypoglycemia in few cases) can mimic many other inborn errors of metabolism such as glycogen storage diseases, lysosomal acid lipase deficiency, lipidosis, citrin deficiency, fatty acid oxidation defect disorders, mitochondrial disorders, and Chanarin–Dorfman syndrome. In recent years, with the application of advanced molecular testing, the diagnosis of these rare diseases can be readily confirmed to initiate effective treatment and prognostic guidance.

In conclusion, GPD1 deficiency is a rare cause of monogenic HTG during infancy, frequently associated with hepatomegaly, hepatic steatosis, and progression to fibrosis or cirrhosis in some cases. This study indicates that *GPD1* deficiency may not be a transient or benign condition, as previously considered, due to the persistence of HTG and liver pathology in a significant proportion of cases. The findings underscore the importance of genetic screening for *GPD1* mutations in patients presenting with these symptoms, which may help guide clinical management and improve diagnostic accuracy. Identifying the predominant variant of GPD1 in any population will probably facilitate rapid molecular analysis by targeting this specific mutation.

## Author Contributions

**A.A-H.** and **M.A.** contributed to study conception, design, conduct, and writing the manuscript. **A.A.** collected the data, and reviewed and edited the manuscript. **S.A.** collected the data, and reviewed and edited the manuscript. All authors contributed to the article.

## Funding

This study was supported by the funding program of Research Chairs (ORF‐RC‐2025‐0500) at King Saud University, Riyadh, Saudi Arabia.

## Disclosure

This work was previously presented as a poster at the ESPGHAN Annual Meeting 2023 of the European Society for Paediatric Gastroenterology Hepatology and Nutrition and published as an abstract in *Journal of Pediatric Gastroenterology and Nutrition*. The abstract is available at https://onlinelibrary.wiley.com/doi/10.1097/MPG.0000000000003823 [17]. All authors approved the submitted version.

## Conflicts of Interest

The authors declare no conflicts of interest.

## Data Availability

All data required to evaluate the conclusions of this study are presented herein. Additional data related to this study can be obtained upon request from the authors.
